# Assessment of quality of life in patients with advanced non-small cell lung carcinoma treated with a combination of carboplatin and paclitaxel*

**DOI:** 10.1590/S1806-37132015000004367

**Published:** 2015

**Authors:** Camila Uanne Resende Avelino, Rafael Marques Cardoso, Suzana Sales de Aguiar, Mário Jorge Sobreira da Silva

**Affiliations:** José Alencar Gomes da Silva National Cancer Institute, Rio de Janeiro, Brazil. Multiprofessional Residence Program in Oncology, José Alencar Gomes da Silva National Cancer Institute, Rio de Janeiro, Brazil; José Alencar Gomes da Silva National Cancer Institute, Rio de Janeiro, Brazil. Chemotherapy Center, Cancer Hospital I, José Alencar Gomes da Silva National Cancer Institute, Rio de Janeiro, Brazil; José Alencar Gomes da Silva National Cancer Institute, Rio de Janeiro, Brazil. Department of Clinical Epidemiology, José Alencar Gomes da Silva National Cancer Institute, Rio de Janeiro, Brazil; José Alencar Gomes da Silva National Cancer Institute, Rio de Janeiro, Brazil. José Alencar Gomes da Silva National Cancer Institute, Rio de Janeiro, Brazil

**Keywords:** Carcinoma, non-small-cell lung, Quality of life, Palliative care, Carboplatin, Paclitaxel

## Abstract

**OBJECTIVE:**

Non-small cell lung carcinoma (NSCLC) is the most common type of lung cancer. Most patients are diagnosed at an advanced stage, palliative chemotherapy therefore being the only treatment option. This study was aimed at evaluating the health-related quality of life (HRQoL) of advanced-stage NSCLC patients receiving palliative chemotherapy with carboplatin and paclitaxel.

**METHODS:**

This was a multiple case study of advanced-stage NSCLC outpatients receiving chemotherapy at a public hospital in Rio de Janeiro, Brazil. The European Organization for Research and Treatment of Cancer Core Quality of Life Questionnaire was used in conjunction with its supplemental lung cancer-specific module in order to assess HRQoL.

**RESULTS:**

Physical and cognitive functioning scale scores differed significantly among chemotherapy cycles, indicating improved and worsened HRQoL, respectively. The differences regarding the scores for pain, loss of appetite, chest pain, and arm/shoulder pain indicated improved HRQoL.

**CONCLUSIONS:**

Chemotherapy was found to improve certain aspects of HRQoL in patients with advanced-stage NSCLC.

## Introduction

For 2015, the estimated incidence of tracheal, lung, and bronchial cancer in the Brazilian population is 27,330 cases.^(^ 1 ^)^ Lung neoplasms are the most common cancers in the world, accounting for nearly 15% of all cancers; the death rate is high, and the 5-year survival rate is less than 15%.^(^ 2 ^)^

Non-small cell lung carcinoma (NSCLC) has the highest incidence of all lung neoplasms, accounting for 80-85% of all cases of lung cancer. ^(^ 2 ^)^ The biology of NSCLC and delayed diagnosis are the main reasons why NSCLC is the leading cause of cancer death worldwide.^(^ 3 ^)^

It is believed that 70% of patients present with advanced disease at diagnosis,^(^ 4 ^)^ and palliative chemotherapy is often indicated.^(^ 5 ^)^ Its goal is to control the signs and symptoms of advanced disease, which can affect the performance status, quality of life, and survival of patients.^(^ 4 ^,^ 6 ^,^ 7 ^)^

Currently, the palliative treatment of advanced-stage (stage IIIB and stage IV) NSCLC frequently involves the use of platinum coordination compounds such as carboplatin in combination with other antineoplastics, such as paclitaxel.^(^ 2 ^)^ Nevertheless, the scientific literature is inconclusive regarding the impact of these drugs on the quality of life of patients with advanced-stage NSCLC,^(^ 6 ^,^ 8 ^)^ which is an underexplored topic in clinical practice. 

Health-related quality of life (HRQoL) can be described as the perception of patients of their own physical well-being, daily activities, psychological well-being, social relations, and disease symptoms.^(^ 9 ^,^ 10 ^)^ Assessment of patients with lung cancer is of great importance because of the increased morbidity and mortality associated with NSCLC.^(^ 11 ^-^ 13 ^)^

Studies have indicated that quality of life assessment is the main predictor of survival, describing it as a relevant outcome in the context of palliative chemotherapy.^(^ 4 ^,^ 13 ^)^ The use of questionnaires and periodic review of HRQoL facilitate communication between the health care team and the patients, optimizing the treatment.^(^ 13 ^)^

The objectives of the present study were to evaluate the HRQoL of advanced-stage NSCLC patients receiving palliative chemotherapy with carboplatin-paclitaxel and to promote a scientific discussion of this issue, which is currently underexplored, particularly in Brazil. 

## Methods

This was a multiple case study with a prospective descriptive analytical design. The study was conducted between May and July of 2013 at the adult chemotherapy outpatient clinic of a public cancer hospital located in the city of Rio de Janeiro, Brazil. The study included advanced-stage lung cancer patients receiving chemotherapy with carboplatin (area under the curve = 4-6) and paclitaxel (175 mg/m^2)^, with a 21-day interval between cycles. Patients with IIIB or IV stage NSCLC were sequentially enrolled in the study. Patients under 18 years of age were excluded, as were those who had undergone chemotherapy less than 5 years prior to the study, those who were diagnosed with a second primary malignancy, those who were unable to answer the questions clearly, and those who were already participating in another research protocol. 

For the evaluation of quality of life, the instruments used were the European Organization for Research and Treatment of Cancer Core Quality of Life Questionnaire (EORTC QLQ-C30) and its supplemental lung cancer-specific module (QLQ-LC13),^(^ 14 ^)^ both of which had previously been translated into Portuguese and validated for use in Brazil.^(^ 12 ^)^ The EORTC QLQ-C30 consists of five functional scales evaluating physical, role, emotional, cognitive, and social functioning; global health status/QoL; three scales measuring symptoms (nausea/vomiting, fatigue, and pain); and 6 items assessing the occurrence and severity of symptoms related to cancer and its treatment. ^(^ 4 ^,^ 11 ^,^ 12 ^)^ The QLQ-LC13 consists of 13 questions regarding the symptoms associated with lung cancer and the most common reactions to the medical treatment of lung cancer. 

All HRQoL scores were calculated in accordance with the rules established by the EORTC.^(^ 9 ^)^ Higher scores on the functional and quality of life scales translated to better HRQoL, whereas higher scores on the symptom scales translated to worse HRQoL. For a better understanding of the results, the symptom scales and items were inverted so that higher scores translated to fewer reports of symptoms and better quality of life.^(^ 15 ^)^

In each chemotherapy cycle, all HRQoL evaluations were used as a unit of analysis. The questionnaires were completed by the patients themselves before the 1st, 2nd, and 4th cycles of chemotherapy in order to compare pre-chemotherapy HRQoL, HRQoL during chemotherapy, and post-chemotherapy HRQoL. When asked to, the interviewer read the questions out to patients. 

We collected data on the following sociodemographic and clinical variables: age; gender; self-reported race; marital status; number of years of schooling (0-7 years or ≥ 8 years); occupation; histological type; clinical stage; performance status; comorbidities; number of drugs used (≤ 4, 5-7, or 8-10); self-reported allergies; family history of cancer; smoking; daily cigarette consumption (< 20 cigarettes/day, low or medium consumption; ≥ 20 cigarettes/day, high consumption); and alcoholism. 

For statistical analysis of the data, we used the IBM SPSS Statistics software package, version 20.0 (IBM Corporation, Armonk, NY, USA). Descriptive statistics included measures of central tendency and dispersion for continuous variables and absolute and relative frequencies for categorical variables. 

In order to assess HRQoL during chemotherapy, we subtracted the mean functional and symptom scale scores for the 2nd chemotherapy cycle from those for the 1st cycle; those for the 4th cycle from those for the 2nd cycle; and those for the 4th cycle from those for the 1st cycle. In order to evaluate the changes in mean scores between cycles, we used the Wilcoxon signed-rank test, the level of significance being set at p < 0.05. In order to interpret the changes in mean HRQoL scores between chemotherapy cycles, we used the criteria proposed by Osoba et al.,^(^ 16 ^)^ changes of 5-10 points in the mean scores being considered small, changes of 10-20 points being considered moderate, and changes of more than 20 points being considered large. 

All ethical principles for research involving human subjects were followed. The study was approved by the Research Ethics Committee of the José Alencar Gomes da Silva National Cancer Institute (Protocol no. CAEE 14472813.9.0000.5274). 

## Results

A total of 18 patients completed the EORTC QLQ-C30 and the QLQ-LC13 for the evaluation of HRQoL before the 1st cycle of chemotherapy. Of those 18 patients, 2 were excluded during the study (1 because of a change in the chemotherapy protocol and 1 because of outpatient treatment discontinuation during the 2nd cycle of chemotherapy), 16 patients remaining in the study. Because of clinical worsening, 3 patients did not receive the 4th cycle of chemotherapy and therefore did not complete the EORTC QLQ-C30 or the QLQ-LC13 for the evaluation of HRQoL. 

The median age of the participants was 63.7 years (mean age, 66 ± 11.1 years), and 56.3% were male. Table 1 shows the sociodemographic and clinical characteristics of the study population. 

**Table 1 -:** Sociodemographic and clinical characteristics of the study participants.

Patient characteristic	n	%
Age, years		
< 65	6	37.5
≥ 65	10	62.5
Gender		
Male	9	56.3
Female	7	43.8
Race		
White	9	56.3
Black	3	18.8
Others	4	25.0
Histological type of NSCLC		
Adenocarcinoma	9	56.3
Others	7	43.8
Stage at diagnosis		
IIIB	6	37.5
IV	10	62.5
Performance status		
1	12	75.0
2	4	25.0
Comorbidities		
Yes	9	56.3
No	7	43.8
Number of drugs used		
< 4	1	6.3
5-7	9	56.3
8-10	6	37.5
Family history of cancer		
Yes	8	50.0
No	8	50.0
History of smoking		
Yes	13	81.3
No	3	18.8
History of alcoholism		
Yes	8	50.0
No	8	50.0

NSCLC: non-small cell lung carcinoma.

Adenocarcinoma was the most prevalent type of NSCLC in the study population, being found in 56.3% of the participants. In addition, 62.5% had stage IV NSCLC. 

Most (75%) of the participants were found to have a performance status of 1 before the 1st cycle of chemotherapy. In addition, nearly 56% had previously diagnosed chronic diseases and were on polypharmacy (≥ 5 different types of drugs). 

Of the 16 participants, 13 (81.3%) declared themselves to be smokers or former smokers and 7 (53.9%) reported smoking at least 20 cigarettes per day. In addition, 50% reported consuming or having consumed alcoholic beverages. 

The mean scores on the EORTC QLQ-C30 functional and quality of life scales were ≥ 59.8. This indicates that the study participants had lower HRQoL scores in the 1st cycle of chemotherapy (Table 2). 

**Table 2 -:** Mean European Organization for Research and Treatment of Cancer Core Quality of Life Questionnaire and European Organization for Research and Treatment of Cancer Core Quality of Life Questionnaire supplemental lung cancer-specific module scores during chemotherapy.

Questionnaire	1st cycle (n = 16)		2nd cycle (n = 16)		4th cycle (n = 13)	
Module/Item	Mean ± SD	Median	Mean ± SD	Median	Mean ± SD	Median
EORTC QLQ-C30						
Functional scale						
Global health status/QoL	67.2 ± 28.0	62.5	73.4 ± 21.6	79.2	77.6 ± 21.9	83.3
Physical functioning	59.8 ± 27.7	60.0	80.4 ± 18.6	83.3	81.5 ± 20.9	93.3
Role functioning	70.7 ± 39.7	91.5	79.2 ± 34.2	100.0	91.0 ± 22.2	100.0
Cognitive functioning	79.0 ± 35.9	100.0	85.4 ± 24.2	100.0	73.1 ± 30.1	83.3
Emotional functioning	63.9 ± 28.6	75.0	73.4 ± 25.5	75.0	66.0 ± 32.2	83.3
Social functioning	91.6 ± 16.3	100.0	96.9 ± 12.5	100.0	91.0 ± 18.8	100.0
Symptom scale						
Fatigue	55.6 ± 36.7	66.7	69.5 ± 27.4	66.7	77.8 ± 20.8	88.9
Pain	60.4 ± 35.4	58.4	80.2 ± 28.0	81.7	78.2 ± 23.9	83.3
Dyspnea	62.5 ± 40.1	66.7	75.0 ± 35.5	100.0	74.4 ± 38.9	100.0
Insomnia	77.1 ± 35.9	100.0	72.9 ± 38.9	100.0	71.8 ± 38.1	100.0
Appetite loss	41.7 ± 46.4	16.5	79.2 ± 40.1	100.0	79.5 ± 39.8	100.0
Nausea/vomiting	91.7 ± 16.1	100.0	89.6 ± 21.0	100.0	92.3 ± 18.8	100.0
Constipation	47.9 ± 50.1	33.4	64.6 ± 44.7	100.0	76.9 ± 43.9	100.0
Diarrhea	100.0 ± 0.0	100.0	91.7 ± 25.8	100.0	92.3 ± 27.7	100.0
Financial difficulties	70.8 ± 41.9	100.0	83.3 ± 32.2	100.0	87.2 ± 25.6	100.0
QLQ-LC13						
Dyspnea	68.8 ± 37.0	83.4	75.7 ± 32.5	88.9	86.3 ± 25.3	100.0
Cough	47.9 ± 40.3	50.0	58.3 ± 35.5	66.7	48.7 ± 44.3	66.7
Hemoptysis	93.8 ± 13.4	100.0	87.5 ± 24.0	100.0	94.9 ± 18.5	100.0
Sore mouth	89.6 ± 29.1	100.0	97.9 ± 8.3	100.0	94.9 ± 12.5	100.0
Dysphagia	87.5 ± 24.0	100.0	93.8 ± 18.1	100.0	82.3 ± 27.7	100.0
Peripheral neuropathy	83.3 ± 29.8	100.0	77.1 ± 39.8	100.0	76.9 ± 39.4	100.0
Alopecia	97.9 ± 8.3	100.0	22.9 ± 33.8	0.0	53.8 ± 51.9	100.0
Chest pain	58.3 ± 46.4	83.4	85.4 ± 27.1	100.0	82.1 ± 37.6	100.0
Arm/shoulder pain	60.4 ± 45.9	83.4	81.3 ± 29.7	100.0	84.6 ± 29.2	100.0
Pain in other body parts	58.3 ± 49.4	100.0	77.1 ± 35.9	100.0	71.8 ± 42.7	100.0

**Figure 1 -:**
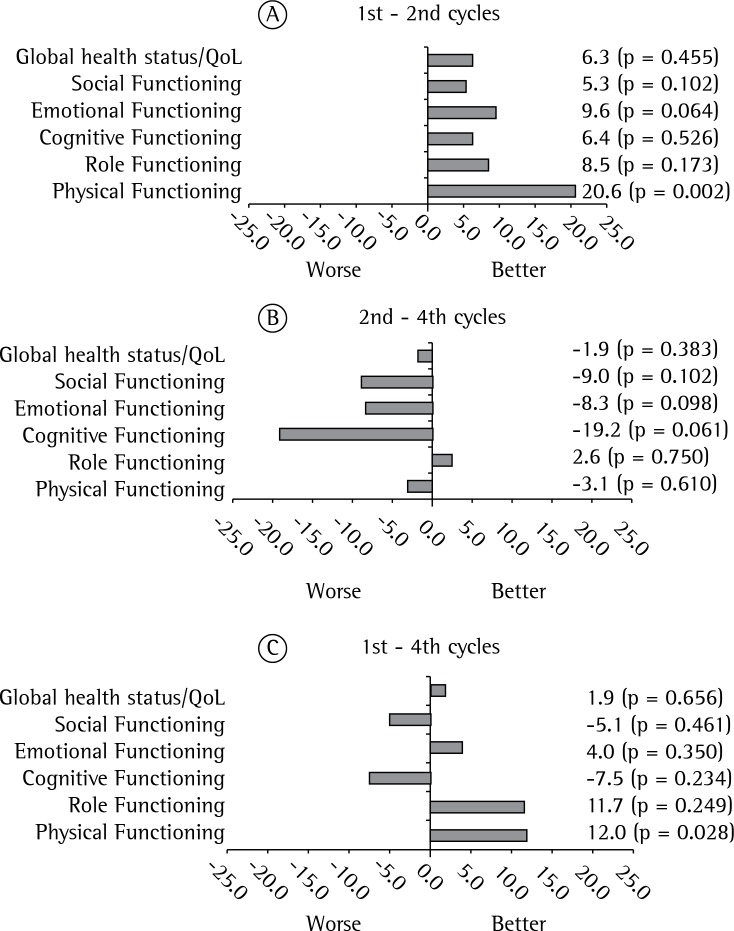
Changes in mean European Organization for Research and Treatment of Cancer Core Quality of Life Questionnaire (EORTC QLQ-C30) functional scale scores. In A, differences in mean EORTC QLQ-C30 scores between the 1st and 2nd cycles of chemotherapy (n = 16); in B, differences in mean EORTC QLQ-C30 scores between the 2nd and 4th cycles of chemotherapy (n = 13); and in C, differences in mean EORTC QLQ-C30 scores between the 1st and 4th cycles of chemotherapy (n = 13). QoL: quality of life.

Figure 1 shows a comparison of the mean EORTC QLQ-C30 functional scale scores in each treatment cycle. There were no significant differences among the scores, the exception being the physical functioning scale scores in the 1st and 2nd cycles (p = 0.002; Figure 1A), showing improved HRQoL in the 2nd cycle of chemotherapy, and in the 1st and 4th cycles (p = 0.028; Figure 1C), showing improved HRQoL in the 4th cycle of chemotherapy. 

Taking into consideration the criteria proposed by Osoba et al.^(^ 16 ^)^ for interpreting the significance of changes in HRQoL scores and the different numbers of patients at each assessment time point, we found a moderate change (of 11.7 points) in the role functioning score between the 1st and 4th cycles of chemotherapy (Figure 1C), a finding that shows a trend toward an improvement in HRQoL. 

There were no changes in the global quality of life/QoL scores between the 2nd and 4th cycles of chemotherapy. However, there was a small change (of 6.3 points) in the global quality of life/QoL scores between the 1st and 2nd cycles of chemotherapy (Figure 1A). 

The symptom scale scores and the scores on the items assessing the occurrence and severity of cancer-related symptoms were higher at the first assessment of quality of life, the exception being the scores for diarrhea. This indicates that the HRQoL of the study participants was worse at that time (Table 2). 

**Figure 2 -:**
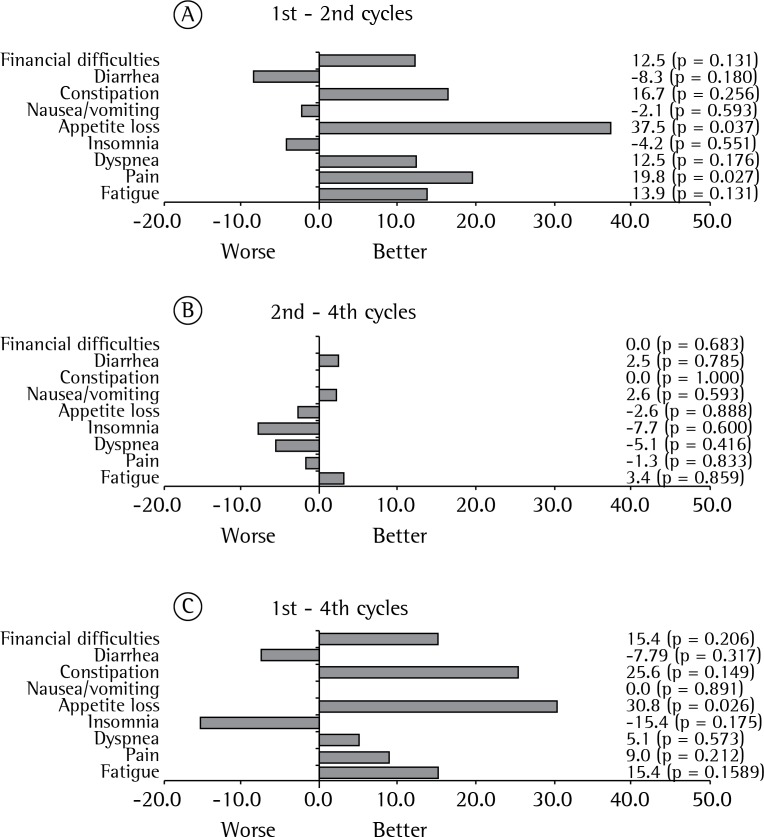
Changes in mean European Organization for Research and Treatment of Cancer Core Quality of Life Questionnaire (EORTC QLQ-C30) symptom scores. In A, differences in mean EORTC QLQ-C30 scores between the 1st and 2nd cycles of chemotherapy (n = 16); in B, differences in mean EORTC QLQ-C30 scores between the 2nd and 4th cycles of chemotherapy (n = 13); and in C, differences in mean EORTC QLQ-C30 scores between the 1st and 4th cycles of chemotherapy (n = 13).

Figure 2 shows a comparison of the mean EORTC QLQ-C30 symptom scores in each cycle of chemotherapy. There was little or no change in the scores during chemotherapy. There were significant differences in pain scores between the 1st and 2nd cycles of chemotherapy (p = 0.027; Figure 2A), as well as in the scores for loss of appetite between the 1st and 2nd cycles (p = 0.037; Figure 2A) and between the 1st and 4th cycles (p = 0.026; Figure 2C). There was a large change in the scores for constipation between the 1st and 4th cycles of chemotherapy (Figure 2C). There were moderate changes in the scores for fatigue, insomnia, and financial difficulties between the 1st and 4th cycles of chemotherapy. These changes suggest an improvement in all of the aforementioned HRQoL aspects except insomnia, which was reported more frequently in the 4th cycle of chemotherapy. 

The QLQ-LC13 scores for dyspnea, cough, sore mouth, chest pain, arm/shoulder pain, and body pain were lower at the first assessment of HRQoL (i.e., in the 1st cycle of chemotherapy). Hemoptysis and alopecia were found to be more common and more severe at the second assessment of HRQoL, whereas dysphagia and peripheral neuropathy were found to be worse in the 4th cycle of chemotherapy. 

**Figure 3 -:**
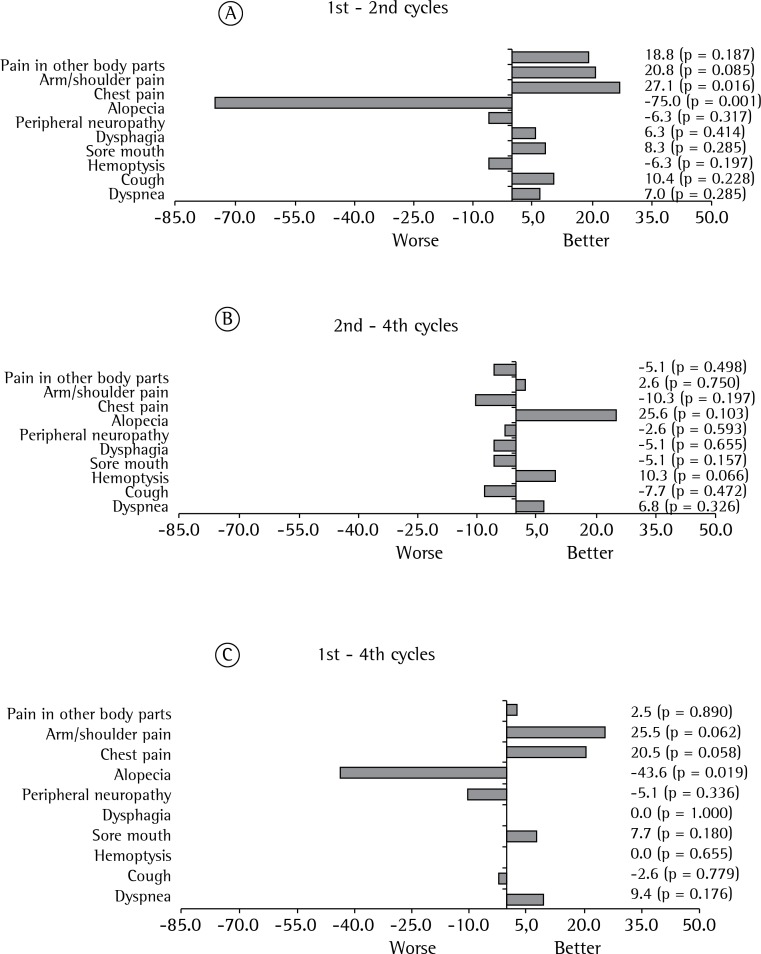
Changes in mean European Organization for Research and Treatment of Cancer Core Quality of Life Questionnaire supplemental lung cancer-specific module (EORTC QLQ-LC13) scores. In A, differences in mean EORTC QLQ-LC13 scores between the 1st and 2nd cycles of chemotherapy (n = 16); in B, differences in mean EORTC QLQ-LC13 scores between the 2nd and 4th cycles of chemotherapy (n = 13); and in C, differences in mean EORTC QLQ-LC13 scores between the 1st and 4th cycles of chemotherapy (n = 13).

Figure 3 shows a comparison of the mean QLQ-LC13 scores in each chemotherapy cycle. There was a significant improvement in chest pain between the 1st and 2nd cycles (p =0.016; Figure 3A). There were significant differences in the scores for alopecia between the 1st and 2nd cycles, as well as between the 1st and 4th cycles. There were moderate changes (of 10.4 and 18.8 points, respectively) in the scores for cough and pain in other body parts between the 1st and 2nd cycles of chemotherapy. These changes indicate an improvement in the aforementioned aspects of HRQoL (Figure 3A). 

## Discussion

In the study population, there was a predominance of White, married, male smokers or former smokers, with stage IV adenocarcinoma. The median age was 63.7 years. Although the logistics and operational aspects of data collection represented a limitation to the selection of study participants, the clinical and sociodemographic characteristics of the participants were consistent with those reported in the literature,^(^ 2 ^,^ 17 ^-^ 21 ^)^ ensuring the external validity of the study. 

The use of structured methods for collecting data and the interpretation of the data brought internal validity to our conclusions, as did the use of multiple sources of evidence and the consistency between such evidence and the results of the study. The possibility of reproducing the present study and the use of statistical analysis brought greater reliability to the study, allowing us to make inferences. Therefore, the present study presents relevant data for the evaluation of clinical oncology patients and raises new hypotheses regarding the possible connections of clinical and sociodemographic variables with the quality of life of patients with advanced-stage NSCLC. 

With regard to the EORTC QLQ-C30 functional scale scores, we noted a trend toward stability at all assessment time points. Wintner et al.^(^ 22 ^)^ stated that chemotherapy alone, regardless of the number of cycles, had no impact on the quality of life of patients with lung cancer. The authors found that the HRQoL scores remained unchanged throughout the treatment period, a finding that is consistent with ours. 

Despite the demonstrated trend toward stability, a significant difference was observed regarding physical function improvement and cognitive function worsening. Braun et al.^(^ 23 ^)^ demonstrated that improvement in physical function was a predictor of survival in patients with lung cancer, confirming that every 10-point increase in physical function is associated with a 10% increase in survival time. However, the changes in the aforementioned aspects might have been influenced by factors such as the use of antineoplastic drugs and drugs for the management of symptoms, as well as by variables such as age, gender, performance status, histological type, stage of the disease, and preexisting comorbidities. 

Grønberg et al.^(^ 24 ^)^ reported that, among NSCLC patients receiving platinum-based chemotherapy, clinical complications appear to be more common in those with severe comorbidities than in those without. Larsson et al.^(^ 25 ^)^ demonstrated significant associations of HRQoL with performance status, age, gender, and disease stage, as well as with EORTC QLQ-C30 and QLQ-LC13 symptom scales and items. Quinten et al.^(^ 26 ^)^ found a correlation between patient-reported physical function and performance status, raising questions regarding the association between self-reported quality of life and the prediction of survival. However, further studies are needed in order to confirm these hypotheses. 

In the present study we found moderate changes in the mean global health status/QoL scores between chemotherapy cycles, with a trend toward improved quality of life, when we used the criteria of Osoba et al.^(^ 16 ^)^ These findings differ from those of Braun et al.,^(^ 23 ^)^ who demonstrated that HRQoL is worse in previously treated patients than in newly diagnosed patients, suggesting that chemotherapy has a negative impact on HRQoL. 

With regard to the most common signs and symptoms experienced by the NSCLC patients investigated in the present study, the results showed an improvement in fatigue, pain, and appetite during chemotherapy, indicating low HRQoL at the first evaluation. Park et al.^(^ 19 ^)^ evaluated the HRQoL of NSCLC patients treated with chemotherapy after a surgical intervention and found no significant changes in fatigue or pain. However, appetite improved during treatment, a result that is similar to ours. Increased loss of appetite has been reported to be associated with shorter survival.^(^ 23 ^)^ Maric et al.^(^ 17 ^)^ reported that although chemotherapy had beneficial effects on fatigue, dyspnea, insomnia, and appetite loss, NSCLC patients undergoing chemotherapy had higher pain scores than did newly diagnosed NSCLC patients. Lin et al.^(^ 27 ^)^ demonstrated that concomitant occurrence and increased severity of the aforementioned symptoms have a negative impact on HRQoL. 

We found no significant changes in the scores for nausea/vomiting and diarrhea when we compared the scores obtained at the first HRQoL assessment with those obtained subsequently. This might be due to the pharmacological characteristics of the drugs in the chemotherapy protocol. Literature data show a low incidence of the aforementioned symptoms when platinum coordination compounds are used in combination with paclitaxel, a factor that should be considered in the choice of drug therapy because it affects the quality of life of patients.^(^ 28 ^)^

Alopecia is a very common side effect of antineoplastic drugs. The study participants reported increased occurrence of alopecia after the 1st chemotherapy cycle, a result that indicates low HRQoL. According to Bonassa and Molina,^(^ 29 ^)^ hair loss is the most devastating effect and can directly affect social and emotional aspects of the quality of life of patients undergoing chemotherapy. 

Our finding of a moderate improvement in cough during chemotherapy is consistent with those of Rolke et al.^(^ 30 ^)^ and Park et al.,^(^ 19 ^)^ who reported that cough tends to improve during chemotherapy. Given that cough negatively influences HRQoL, there is a need for therapeutic interventions for the management of this symptom.^(^ 31 ^)^

Rapid detection of the emergence or worsening of a sign or symptom through periodic assessment of HRQoL allows therapeutic interventions to be performed in a more immediate way, optimizing the treatment of cancer patients and, consequently, impacting their survival.^(^ 10 ^,^ 13 ^)^ However, the assessment of quality of life in daily clinical practice is little discussed in the literature, despite its recognized importance for monitoring the disease and improving communication between the health care team and the patient.^(^ 10 ^,^ 23 ^,^ 32 ^)^

The present study explored self-reported quality of life in advanced NSCLC patients receiving chemotherapy, with the objective of gaining a better understanding of how chemotherapy with carboplatin and paclitaxel influences HRQoL. The importance of patient perception of their own health is highlighted within the context of the complexity of cancer, which is a disease that affects every dimension of life and the way in which individuals perceive the environment, the diagnosis, and the therapy.^(^ 10 ^)^ Therefore, the combination of periodic quality of life assessments and clinical practice should be more extensively discussed in the scientific literature in order to improve the understanding of aspects that define patient health and the benefits arising from it. Although the changes in HRQoL scores between chemotherapy cycles were small, chemotherapy was found to improve the HRQoL of the study participants, having a greater impact on physical and cognitive functioning and on cancer-related symptoms such as pain and loss of appetite. 

With regard to the state of the art, the present study can be considered innovative because it provides elements that are essential to the assessment of quality of life in clinical practice. Further studies should be conducted in order to evaluate the association of sociodemographic and clinical variables such as polypharmacy and comorbidities with aspects of the quality of life of patients undergoing chemotherapy. Because of their extensive knowledge of drugs and their toxicity profile, pharmacists should be involved in studies of quality of life assessment, analyzing the connection between drug therapy and the severity of signs and self-reported symptoms, given their impact on certain aspects of HRQoL. 
